# Effects of feeding earthworm or vermicompost on early life performance of broilers under challenging dietary conditions

**DOI:** 10.1016/j.psj.2024.104341

**Published:** 2024-09-18

**Authors:** Gürbüz Daş, John M. Brameld, Tim Parr, Mohammad M. Seyedalmoosavi, Solvig Görs, Cornelia C. Metges

**Affiliations:** ⁎ Research Institute for Farm Animal Biology (FBN), Dummerstorf, 18196, Germany; † School of Biosciences, University of Nottingham, Sutton Bonington Campus, Loughborough, Leicestershire LE12 5RD, UK

**Keywords:** challenge diet, bacterial DNA, fecal host DNA, non-starch polysaccharide, pasty vent

## Abstract

We investigated if feeding earthworms (**EW**) or vermicompost (**VC**) to broilers improves performance and aids in coping with dietary challenges from a soluble non-starch polysaccharide (**NSP**)-enriched diet (negative control diet; **CON-**). Newly-hatched male Cobb-500 birds (N = 480) were fed either a positive (+) control diet (**CON+**, n = 240) or CON+ supplemented with either 1% EW (**CON+EW**; n = 120) or 1% VC in DM (**CON+VC**; n = 120) for 8 d (Period 1; **P1**). At the end of P1, blood and intestinal samples were taken from half the birds in each group. Half of remaining birds on CON+ stayed on CON+ for further 8 d (**P2**; d9–16) or switched to **CON-**. Birds on CON+EW and CON+VC in P1 were switched to CON- in P2 (**CON-EW** and **CON-VC**, respectively). The CON+VC improved (*P* < 0.05) BW and ADG in P1 through an elevated feed intake (**FI**) (*P* < 0.05) with no effect on FCR. CON+EW did not differ from the CON+ in terms of growth and FI in P1. In P2 CON- did not affect growth or DMI relative to CON+. In the end of P2, 10% of CON+ birds had pasty vent (**PV**). CON- increased incidence of PV and CON-VC aggravated this effect (*P* < 0.05), whereas CON-EW did not differ from CON+. CON- diet reduced proportion of 16S rDNA in colon digesta (*P* = 0.049), while CON-EW and CON-VC did not differ from CON+. Compared to CON-, CON-EW tended to decrease (*P* = 0.072) incidence of PV. Ceca were heavier (*P* < 0.05) in CON-EW than in CON+ fed birds. In conclusion, the challenge diet induced PV and reduced bacterial 16S rDNA in colon digesta, likely due to soluble NSP-induced anti-nutritive effects. VC supplementation enhanced early growth by increasing feed intake. Provision of EW did not impact performance but decreased incidence of PV and increased cecal size, suggesting that potential inoculation with beneficial microorganisms may counteract NSP effects.

## INTRODUCTION

In modern broiler production, the chicks hatch in artificial incubators and are reared under environmental conditions with high hygienic standards, potentially implying the lack of a proper early exposure to microorganism, particularly those with beneficial effects (Zenner et al., 2021). An important environmental factor contributing to the transmission of microorganisms from preceding flocks to newly hatched chicks is the litter used in the housing environment. This serves as an important inoculum for the establishment of gastrointestinal microbiome (Oakley et al., 2014), even under high biosecurity standards when the litter is removed and the houses are cleaned, disinfected, fumigated and given a break. In addition, microorganisms in the host diet may be considered as a contributor to microbial development in the gastrointestinal tract (**GIT**) of the young chicks (Diaz Carrasco et al., 2019), as similarities between diet microbiota and intestinal microbiota in young chicks have been demonstrated (Olson et al., 2020). Early microbial colonization of the GIT plays a crucial role in immune system development, which is vital for pathogen control (Zenner et al., 2021). Broilers, especially those with rapid growth rates, face difficulties in overcoming physiological, behavioral, and immunological challenges, as highlighted by Koenen et al. (2002) and Rauw (2012). This impaired ability of the birds to overcome such challenges could be at least partly related to the lack of hen-chick interactions that would allow the acquisition of initial gut microbiota from the mother or by the mother from the environment. In maternal care (i.e., natural brooding), the mother hen attracts chicks to profitable feeds while diverting their attention from harmful or unprofitable feeds (Nicol and Pope, 1996; Edgar et al., 2016). Besides insects, earthworms (**EW**) are natural feed sources of animal origin for avian species, including chickens. As EW rely on microorganisms to digest cell wall components of plant materials and have a relatively high microbial activity in their gut (Curry and Schmidt, 2007), they may be considered as microbiota inoculates for chicks when fed at early life stages.

More than half of 8,300 well-segmented worm species (i.e. Oligochaetes) are terrestrial EW. In contrast to anecics (burrowers) and endogeic species (soil feeders), epigeic EW species have the potential to be used as nutrient transformers as they are litter dwellers, which consume large amounts of coarse particulate organic matter such as decomposed litter and excrete holorganic fecal pellets, often referred to as vermicompost (**VC**) (Dominguez and Edwards, 2011). Among the epigeic species, only 5 EW species have been identified as suitable for vermicomposting under human care (Curry and Schmidt, 2007; Dominguez and Edwards, 2011). The protein content of EW meal (58–66% of DM) is comparable to that of fishmeal (61%), which is also a protein source of animal origin. Compared with fishmeal, the amino acid (**AA**) composition of EW meal is particularly high in arginine and lysine but low in methionine (Reinecke et al., 1991). In terms of essential amino acid composition, earthworm meal has higher levels of arginine, histidine, leucine, lysine, methionine, and threonine but lower concentrations of isoleucine, phenylalanine, and valine compared to soybean meal (Chiu et al., 2016). As shown earlier by Taboga (1980), there was no difference between growth rates of chickens fed on a protein‐free diet supplemented with EW and vitamins instead of a control diet. Considering the high fraction of human edible ingredients (> 80%) currently used in poultry diets that lead to feed-food competition (Mottet and Tempio, 2017), the use of alternative proteins and fiber-rich diets may be a better option in the future (Van de Weerd et al., 2009). Non-starch polysaccharides (**NSP**) constitute the primary portion of dietary fiber. Chickens lack endogenous digestive enzymes required to break down NSP in the GIT, rendering them inherently indigestible. Depending on the fermentation characteristics of NSP, they can be partially utilized by the microorganisms in the lower GIT (Englyst, 1989; Bach Knudsen, 2001). It is well known that soluble NSP (i.e., highly fermentable) induce anti-nutritive effects in poultry mainly through increased viscosity (Bach Knudsen, 2001). Therefore, exogenous sources of enzymes and microorganisms that can contribute to NSP degradation can have favorable effects on the overall performance and health of broilers. According to Fujii et al. (2012), the EW gut contains microorganisms with exocellulase and xylanase activities, which may be associated with the lignocellulose decomposition activity of EW under natural circumstances. In addition, Spencer et al (1998) documented that broilers fed VC were less frequently contaminated with *Salmonella typhimurium* in the crop and cecum, suggesting potential health promoting effects of EW and VC. Given their potential to serve as microbiota inoculants that may help degrade NSP and mitigate their anti-nutritive effects, we hypothesized that feeding EW or VC to broiler chicks early in life would aid in overcoming dietary challenges and thus enhance health and performance. The objective of this study was to assess potential benefits of feeding EW or VC to broiler chicks under nutritionally challenging conditions, specifically when fed a diet enriched with NSP, which may negatively impact broiler performance and health.

## MATERIALS AND METHODS

Ethical approval for the experiment was obtained from the State ethics committee for animal experimentations (Mecklenburg-Western Pomerania State Office for Agriculture, Food Safety and Fisheries, Germany; permission no.: 7221.3-2-015/19-1). The experiment was conducted in accordance with EU animal welfare rules (animal care and handling, stunning, slaughter) and all sampling procedures were performed by trained staff.

### Animals and Experimental Design

**Figure 1:**
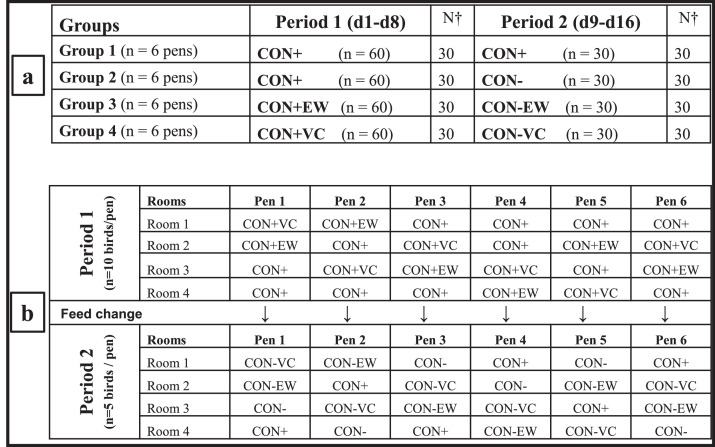
Experimental groups and diets fed to the birds in relation to 2 feeding periods (A), and randomized allocation of the birds to the experimental diets offered in pens of different rooms with respect to feeding periods in a single batch (B). Total number of birds used in 2 batches was N = 480. Abbreviations: CON+: positive control diet; CON-: negative control diet; CON+EW: positive control diet plus 1% of earthworm; CON+VC: positive control diet supplemented with 1% vermicompost; CON-EW: negative control diet plus 1% earthworm; CON-VC: negative control diet supplemented with 1% vermicompost. N^†^: Number of birds killed at the end of each period in each batch.

A total of 480 male broiler chicks (Cobb-500) was used in a feeding experiment with 2 identical batches (**B**) each with 240 birds. The sexed chicks were purchased from a commercial hatchery (Cobb Germany Avimex GmbH, Brüterei Wiesenena, Wiedemar, Germany), where they received vaccinations against Infectious Bronchitis and Newcastle Disease. Immediately after arrival of the birds, they were weighed and allocated to one of 4 feeding groups. A detailed presentation of the experimental design and feeding groups in relation to dietary treatments and time periods, as well as allocation of the groups to rooms and pens in a single batch are summarized in Figures 1A and 1B. The 4 groups were distributed across 4 rooms, with each room having 6 pens, and each pen containing 10 birds at the start of the experiment (Figure 1B). For each batch and starting from the first d of life onwards, the birds were fed either a corn-soybean-meal-based positive control diet (**CON+**, n = 120 birds in 12 pen replicates) or CON+ supplemented with either 1% earthworms (**CON+EW**; n = 60 birds in 6 pen replicates) or 1% vermicompost (**CON+VC**; n = 60 birds in 6 pen replicates) in dry matter (**DM**) for 8 d (Period 1; **P1**: d1 - 8). At the end of P1, half of the birds from each group were euthanized and sampled for blood, digesta and intestinal size measurements. Half of the remaining birds on the CON+ diet (n = 30 birds in 6 replicate pens) were kept on the same positive control diet (i.e., CON+) for further 8 d (**P2**: d9–16), while the other half (n = 30 birds in 6 replicate pens) were switched to a challenge diet, that is, a negative control diet (i.e., **CON-)** in P2. The CON- replaced approximately 50% of corn in the CON+ with wheat, barley and rye (Table 1) to produce the challenge diet with a higher amount of soluble NSP (Table 2). The birds consuming EW and VC on top of the CON+ diet (i.e., CON+EW and CON+VC) in P1 were then switched to the CON- diet (i.e., **CON-EW** and **CON-VC**, respectively). On d 16, all remaining birds were euthanized for further sampling and the experiment was terminated. The group-specific diets and drinking water were provided ad libitum in both P1 and P2.

**Table 1:** Ingredients and analyzed chemical composition of the experimental diets and earthworms (**EW**).

Item	CON+	CON-	CON+VC	CON-VC	EW
Ingredients, g/kg as fed					
Corn	580.85	278.85	533.80	229.70	-
Soybean meal (480 CP)	349.00	334.00	354.00	340.00	-
Soybean oil (8,957 kcal)	25.00	41.00	40.00	57.00	-
Wheat	0.00	100.00	0.00	100.00	-
Barley	0.00	100.00	0.00	100.00	-
Rye	0.00	100.00	0.00	100.00	-
Vermicompost^1^	0.00	0.00	28.25	28.25	-
Premix^2^	5.00	5.00	5.00	5.00	-
Lime fine	14.80	14.80	13.40	13.20	-
Monocalcium phosphate	14.40	14.00	14.40	14.40	-
NaCl	1.90	1.90	1.90	1.90	-
NaHCO_3_	2.80	2.80	2.80	2.80	-
L-Lysine HCl	2.00	2.45	1.95	2.40	-
DL-Methionine	3.15	3.40	3.25	3.45	-
L-Threonine	0.80	1.15	0.85	1.15	-
L-Valine	0.30	0.65	0.40	0.75	-
Chemical analysis, g/kg DM					
Dry matter, g/kg	890	894	882	887	145
Crude protein	242	243	247	251	630
Crude fat	57	67	72	80	62
Starch^3^	457	418	430	385	22
Sugars	38	43	41	43	18
Crude fiber	22	27	32	39	22
NDF	126	164	130	167	63
ADF	53	52	49	54	52
Crude ash	67.5	66.0	68.5	69.5	83
Ca	11.8	10.0	10.4	10.5	7.1
P	7.6	7.2	7.2	8.0	9.4
Mg	2.0	1.9	2.1	2.1	2.1
K	7.6	10.9	11.1	10.9	11.3
Na	1.7	1.8	2.0	1.9	6.1
Metabolizable energy					
ME, kcal/kg DM	3,298	3,251	3,346	3,251	2,980

Each value represents average of 2 analyzed samples.

1 DM of vermicompost (**VC**) was 35.4%. In order to ensure 1% VC in the DM of the supplemented diets, 28.25 g VC (as is) was included in 1,000 g of the supplemented diets.

2 Contents per kg premix: 2,000,000 I.U. Vit. A (retinol acetate); 500,000 I.U. Vit. D3 (cholecalciferol); 10000 I.U. Vit. E (α-tocopherol acetate); 300 mg Vit. K_3_ (menadione); 400 mg Vit. B1; 1,500 mg Vit. B2; 700 mg Vit. B6 (pyridoxine-HCL); 4,000 μg Vit. B12; 7,000 mg niacin; 40 mg biotin; 200 mg folic acid; 2,400 mg pantothenic acid (D-pantothenic acid); 920,000 mg choline chloride; 16,000 mg iron; 12,000 mg zinc; 17,000 mg manganese; 2,400 mg copper; 160 mg iodate; 30 mg selenium.

3 For earthworms it is glycogen.

**Table 2:** Sugars and sugar acids as constituents of soluble- and insoluble non-starch polysaccharides (**NSP**), as well as lignin and dietary fiber contents of the experimental diets (g/kg DM feed).

	Experimental diets			
g / kg DM	CON+	CON-	CON+VC	CON-VC
Rhamnose	0.9	0.9	0.9	1.0
Fucose	1.1	1.2	1.2	1.2
Arabinose	20.5	22.6	18.9	22.5
Xylose	18.9	23.3	15.6	23.7
Mannose	5.1	6.4	5.0	6.8
Galactose	18.5	19.4	19.9	19.1
Glucose	31.7	37.3	28.3	37.5
Uronic acid	14.9	13.9	14.8	14.6
Soluble NSP	19.0	25.5	21.4	27.4
Insoluble NSP	92.7	99.4	83.2	99.0
Total NSP	111.7	124.9	104.6	126.4
Klason lignin	11.7	13.9	17.6	29.8
Dietary fiber^1^	123.4	138.8	122.2	156.3

Abbreviations: CON+: positive control diet; CON-: negative control diet; CON+VC: positive control diet supplemented with 1% vermicompost; CON-VC: negative control diet supplemented with 1% vermicompost in dry matter.

Each value represents average of 2 samples.

1 Dietary fiber represents the sum of total NSP and lignin.

The climatic conditions that prevailed in the rooms were controlled by an automatic system to ensure uniform temperature, light and ventilation conditions in the pens within and between rooms and corresponded to the recommendations for the commercial rearing of broilers. Wood shavings were used as litter material, which were placed on the floor of the individual pens in equal quantity (i.e., 1,600 g/pen). Litter DM content of a well-mixed fresh litter sample was determined at the beginning of experiment (97.44%). At the end of P1 and P2, each pen was sampled for a representative litter sample to determine DM contents by oven-drying at 103°C until a constant weight was achieved.

### Nutrient Analysis and Microbiological Assessment of the Experimental Diets

Ingredients and nutrient contents of the 4 main diets and EW are given in Table 1. All 4 diets had similar energy and crude protein contents, and were in line with the nutrient recommendations for the broiler genotype used in this experiment (Cobb500 Broiler Performance and Nutrition Supplement, 2022). The diets were manufactured by a company, Research Diet Services BV, Wijk bij Duurstede, The Netherlands. No NSP degrading enzymes were added to the diets. The diets were not heated for pelleting, and were given to the birds in mash form. Representative feed and EW samples were collected regularly in both experimental batches, and stored at −20°C for chemical analyses. In each batch, the feed samples were then pooled per diet, and a representative sample of each diet was analyzed for DM content, crude ash, crude protein (**CP**), crude fat, starch, crude fiber (**CF**), neutral detergent fiber (**NDF**), acid detergent fiber (**ADF**), and selected macro- and trace minerals (Table 1) by the accredited feed laboratory of Landwirtschaftliche Untersuchungs-und Forschungsanstalt, LMS Agrarberatung GmbH (Rostock, Germany) using standard methods (VDLUFA, 2022a). Soluble (**S-**) and insoluble NSP (**I-NSP**) as well as their constituent sugars and sugar acids together with lignin were measured in the diets at the Dept. of Animal Science, Aarhus University, Denmark, as described (Bach Knudsen and Lærke, 2018), and are summarized in Table 2. The AA compositions of the diets, EW and VC samples were analyzed using high performance liquid chromatography (**HPLC**) (1,200/1,260 Infinity II series, Agilent Technology, Waldbronn, Germany) as described by Kuhla, et al. (2010) after acidic hydrolysis of samples. Five mg of lyophilized ground sample was suspended in 2 mL of 6 M HCl. After addition of 50 µL of ascorbic acid (16 mg/mL ultrapure water), oxygen was removed from the suspension with a strong N_2_ flow for 1 min, and then the sample was heated for 22 h at 110°C. The hydrolysate was dried at 60°C under N_2_, re-suspended in 2 mL of 0.1 M HCl, and then centrifuged at 1,573 × *g* at 4°C for 20 min. For AA analysis the supernatant was diluted 1/10 with ultrapure water. The AA chromatograms were integrated with the OpenLab ChemStation software (Agilent Technologies, Waldbronn, Germany) and the AA concentrations were calculated based on a calibration with a standard AA mixture (A9906, Sigma-Aldrich/Merck, Darmstadt, Germany). The AA compositions (mg/g DM) of the diets and EW are summarized in Supplementary Table 1. The microbial quality of feed and EW samples were assessed by an accredited laboratory using standard methods (VDLUFA, 2022b), and a summary of the assessment is presented in Supplementary Table 2.

### Provision of Earthworms to the Birds

The EW used in this experiment belong to the European nightcrawler, that is, *Dendrobaena veneta* (sometimes referred to as *Eisenia hortensis*). The EW were provided by an EW rearing company (Martin Langhoff SUPERWURM e.K., Düren, Germany). Based on the feed intake (**FI**) of EW consuming group (i.e., CON+EW in P1 and CON-EW in P2) from the previous day, the amount of EW to be given the next day were determined. For this purpose, daily DM intake (**DMI**) of CON+EW (average of 6 pens) in P1 (i.e. CON-EW in P2) from the previous day was calculated based on feed and EW consumption. Earthworms were then given to the birds of CON+EW or CON-EW in addition to their ad libitum feed as 1% of total DMI from the previous day. The amount of EW (given as fresh substrate) corresponded to 6.7% of total fresh matter intake of the previous day. All 6 pens of CON+EW and CON-EW received the same amount of EW on the same day of P1 and P2, respectively. On each day the birds were provided EW, the EW were separated from the rearing soil material, weighed and offered to the birds on a feeding plate (diameter of ca. 30 cm). Small-sized EW were used for the study. The overall average weight of EW used in the study was 157 mg per worm (SD = 47; n = 36 batch measurements on randomly selected batches of 13 - 37 worms each). Time spent by the birds consuming EW (n = 6 pens for both CON+EW and CON-EW) were assessed in the second batch only. For this purpose, the time at which EW were brought into the pens was recorded. Thereafter, the EW receiving pens were observed frequently and the time at which all the EW were eaten by the birds determined.

### Performance Measurements, Host and Microbial DNA, Intestine Size and Humoral Immunity

The birds were weighed on the day of arrival and the average initial BW was determined. Pen based FI and individual BW were measured at daily and weekly intervals, respectively. Pen based average BW, daily weight gain (**ADG**), FI, DMI via feed and EW intake, and feed conversion ratio (**FCR**, i.e., feed:gain ratio, g:g) were calculated at the end of each period of each batch, after correcting for mortality. Heterogeneity in bird growth was assessed with coefficient of variation in BW of the birds in each pen (**CV in BW**, %). During the last 6 d of P2, all birds were evaluated daily for the incidence of pasty vent (**PV**) by visual inspection based on the presence and absence of sticky feces in the vent area as described by De Cesare et al. (2017). Mortality was recorded daily. On d 8 and 16 (i.e., end of P1 and P2, respectively) of B2, randomly selected animals (n = 15 birds per group) were weighed and slaughtered after electrical stunning for blood collection, intestinal size measurements and colon digesta sampling.

Individual colon digesta samples (200 mg/bird) were collected from 2 to 3 birds of each pen at slaughter (d8 and d16) for quantification of host DNA and bacterial 16S genomic rDNA in colon contents in the second batch of the experiment. Briefly, colon contents were snap frozen using liquid nitrogen, stored at -80°C and then shipped on dry ice to the University of Nottingham, where DNA extraction and quantitative PCR were performed using a modified protocol originally developed for pig feces (Slinger et al., 2019). Unfortunately, the modified phenol chloroform procedure used for extraction of DNA from pig feces (Slinger et al., 2019) had been found not to work as well for chicken excreta (Slinger et al., 2020). Hence chicken colon content DNA was extracted using a protocol that employed bead-beating and the QIAamp DNA Stool Mini Kit (Qiagen) according to the manufacturer's instructions. Briefly, 200 mg of colon content was homogenized in MagNA Lyser Green Bead Tubes (Roche) in the presence of Buffer ASL (Qiagen) and then centrifuged. Supernatant was processed using the QIAamp DNA Stool Mini Kit (Qiagen) and the resulting DNA was assessed by NanoDrop 2000 (Thermo Scientific), then stored at -20°C prior to quantitative PCR. To assess DNA composition, the SYBR Green (Roche) quantitative PCR methodology was used. Primers for detecting host (chicken) DNA were designed specifically to the chicken *cytochrome B* gene (CYTB, Accession no. NP_006926) [Forward primer: 5′ACTCATAGCCACCGCCTTTG3’; Reverse primer: 5′AAGGGTTGGGTTGTCGACTG3’], which has a high copy gene, and is thus expected to be abundant in colon content, even if DNA in feces is expected to be degraded (Slinger et al., 2019;^2020^). For the detection of total bacterial 16S rDNA, published universal primers were used (Mieszkin et al., 2009; Forward primer: 5′CGGTGAATACGTTCCCGG3’; Reverse primer: 5′TACGGCTACCTTGTTACGACTT3’). Quantitative PCR was performed on a LightCycler 480 (Roche) instrument to assess the DNA composition of the colon content. DNA samples diluted to 5 ng/μl were used to create DNA pools that were used to produce a 1:4 dilution series to generate standard curves and relative proportions (i.e., y / 1; where y = the target DNA and 1 is the total DNA) of chicken CYTB DNA and bacterial 16S rDNA determined. Colonic DNA originating from other organisms (e.g., plant DNA in feed and EW DNA) was not considered.

The total weights of the small intestine and ceca were also measured. For the small intestines only jejunum and ileum weights were recorded together, while the duodenum was excluded, as it was not possible to separate the pancreas precisely in young birds due to time constraints during slaughter. The residual egg-yolk-sac attached to the small intestine at Meckel's diverticulum was not removed and weighed together with jejunum and ileum (**JI-ResEYS**). Ceca were cut from the junction point with the ileum and colon and the full weight of the cecum pairs was determined collectively.

Slaughter blood was collected at the end of the 2 periods of the second batch (n = 15 birds / group in each period) using K3-EDTA tubes (Sarstedt AG & Co., Nümbrecht, Germany). Plasma was harvested after centrifugation and stored at -20°C for later use. Commercial ELISA kits (IgY: Kit No. E30-104; IgM: Kit No. E30-103; IgA: Kit No. E30-102; Bethyl Laboratories, Inc, Montgomery, TX) were used to determine concentrations of immunoglobulin isotypes (**IgY, IgM, IgA**) in plasma samples. Plasma albumin and total protein concentrations were measured with ABX Pentra 400 using commercial kits [albumin: Kit No. A11A01664, total protein: Kit No. 553-412 (MTI diagnostics, Idstein, Germany)]. The globulin concentration was then calculated as total protein minus albumin.

### Statistical Analyses

The performance data were analyzed with analysis of variance using the GLM and Mixed procedures of SAS (V9.4) (SAS Institute Inc., 2022). Data from each period were analyzed separately using different models for pen-based measurements and bird-individual measurements. The experimental unit was a pen for growth (e.g., average bird weight in a pen, average ADG of birds in a pen, FCR), feed intake and litter moisture parameters. Similarly, percentage of animals with PV and time spent consuming EW were calculated based on pen data. Because PV was measured over the last 6 d of P2 on a daily basis for birds in each pen, average percentage of birds with PV during the last 6 d of P2 was calculated for each pen (% birds with PV across 6 d). For analysis of intestinal size and plasma immunoglobulin isotype measurements, the bird-individual measurements were used (i.e., the replicate was a bird). For all the pen-based data, total number of replicate pens throughout the 2 batches was N = 48 pens. For most groups, this consisted of n = 6 pens per diet per batch in both P1 and P2 (see Figure 1), but n = 12 pens per batch for the CON+ diet in period 1.

The statistical model for pen based variables included fixed effects of diet, batch, diet × batch and blocking effects of room plus residual random error. For the bird-individually measured data, the statistical model was the same as for pen data, but additionally included blocking effects of pens. For the time spent consuming worms, the statistical model included only day and room effects with pen as repeated subject over time in an autoregressive (AR(1)) covariance structure using Proc Mixed of SAS (V9.4). No batch effect was included in this model, because data were only available from the second batch.

Least square means (**LSMEANS**) of groups were separated using the Tukey test. Group differences were considered significant at *P* < 0.05, and tendency to differ was declared at 0.05 < *P* ≤ 0.10. Data are presented as LSMEANS and their standard errors (**SE**). In the interest of a clear presentation in the tables, only the most conservative SE (i.e. the largest) of LSMEANS is presented. As the numbers of replicates for all groups were the same in the second period (n = 6), SEs of LSMEANS were identical for this period.

The relative proportion of host and bacterial genomic DNA data showed non-normal distributions (i.e., Kolmogorov-Smirnov test, *P* < 0.05), and the distributions did not improve after logarithmic transformation (i.e., Kolmogorov-Smirnov test, *P* < 0.05). Thus the genomic DNA data were analyzed using a non-parametric Kruskal–Wallis test with Monte Carlo estimates of the exact *P-*values. If a significant effect of diet on host or bacterial DNA was found in the 4 groups using the Kruskal-Wallis test, the Dunn's test (alpha = 0.05) was used as a post-hoc test for multiple comparisons, as suggested by Morison (2002). The Dunn's test was performed with the aid of a SAS macro, developed by Elliott and Hynan (2011) and implemented in the NPAR1WAY procedure of SAS (V9.4). In order to test whether host and bacterial DNA in colon contents are associated with feed utilization efficiency, correlations between FCR, DMI and genomic DNA of host and bacteria were calculated. For this purpose, pooled data across all groups were analyzed for each period separately. The experimental unit for the correlation analysis was the pen. Although each pen had a single DMI and FCR value, there were 2 to 3 birds from each pen that were sampled for DNA measurements. In order to include the DNA data in the correlation analysis, DNA results from 2 to 3 birds of the same pen were then pooled by pen.

## RESULTS

### Mortality, Growth, Feed Intake and Feed Conversion Ratio

The overall mortality across the groups, 2 periods and 2 experimental batches was 1.1%. As overall mortality was low, no statistical comparison was made between the groups (Table 3). Nevertheless, most of the dead birds (7 out of 10) in P1 and P2 belonged to the CON+ diet. None of the treatments influenced homogeneity in BW of the birds (i.e. CV in BW) in either period (*P* > 0.05). The treatment effects resulted in significant differences in ADG, WG and BW of the birds consuming different diets in P1 (*P* < 0.05; Table 3). As compared to CON+ diet, CON+VC improved (*P* < 0.05) ADG, WG and BW in P1, through an elevated feed intake (*P* < 0.05) with no effect on FCR (*P* > 0.05). The CON+EW did not differ from the CON+ in terms of growth and feed intake parameters (*P* > 0.05) in P1, whereas CON+VC tended to (*P* = 0.06–0.103) result in higher growth and feed intake parameters than did CON+EW in P1. The DMI through feed consumption tended to be higher (*P* = 0.099) in CON+VC than in CON+ or CON+EW. However, the total DMI through feed and EW intake did not differ significantly between the 3 groups in P1 (*P* = 0.121).

**Table 3:** Mortality, heterogeneity in growth, pen-based average growth, feed intake, feed, earthworm and dry matter intakes, and feed conversion efficiency estimates for broilers receiving different diets in 2 consecutive periods of 8 d each.

	Diets					*P-values*		
Period 1 (d1-8)	CON+	CON-	CON+EW	CON+VC	*SE*	*Diet*	*Batch*	*DxB*
Mortality, %	2.5	n.a.	0.0	1.7	*-*	*-*	*-*	*-*
CV in BW, %	13.5	n.a.	11.9	11.2	*1.06*	*0.188*	*0.393*	*0.239*
BW, g/bird	192^a^	n.a.	193^ab^†	203^b^†	*2.97*	*0.019*	*0.001*	*0.926*
WG, g/bird	151^a^	n.a.	153^ab^†	162^b^†	*2.90*	*0.022*	*0.028*	*0.957*
ADG, g/bird	18.9^a^	n.a.	19.1^ab^†	20.2^b^†	*0.36*	*0.022*	*0.028*	*0.957*
Feed intake, g/bird	176^a^	n.a.	176^ab^‡	186^b^‡	*2.94*	*0.025*	*0.208*	*0.764*
FCR_1, g:g	1.16	n.a.	1.15	1.15	*0.019*	*0.807*	*0.001*	*0.838*
DMI_feed, g/bird	161	n.a.	161	168	*2.69*	*0.099*	*0.088*	*0.775*
DMI_EW, g/bird	0.00	n.a.	1.22	0.00	*-*	*-*	*-*	*-*
Total DMI, g/bird	161	n.a.	162	168	*2.69*	*0.121*	*0.086*	*0.770*
FCR_2, g:g	1.07	n.a.	1.06	1.04	*0.018*	*0.483*	*0.001*	*0.824*
**Period 2 (d9-16)**	**CON+**	**CON-**	**CON-EW**	**CON-VC**				
Mortality, %	1.7	0.0	1.7	0.0	*-*	*-*	*-*	*-*
CV in BW (%)	14.8	10.8	10.6	11.6	*1.59*	*0.239*	*0.573*	*0.507*
BW, g/bird	549^a^	580^ab^	578^ab^	600^b^	*11.63*	*0.036*	*0.001*	*0.856*
WG, g/bird	360	385	385	397	*10.02*	*0.084*	*0.004*	*0.829*
ADG, g/bird	45.0	48.1	48.1	49.6	*1.25*	*0.084*	*0.004*	*0.829*
Feed intake, g/bird	465	494	483	501	*10.69*	*0.106*	*0.062*	*0.819*
FCR_1 (g:g)	1.30	1.29	1.26	1.27	*0.030*	*0.806*	*0.099*	*0.967*
DMI_feed, g/bird	425	454	443	456	*9.77*	*0.122*	*0.116*	*0.806*
DMI_EW, g/bird	n.a.	n.a.	3.89	n.a.	*-*	*-*	*-*	*-*
Total DMI, g/bird	425	454	447	456	*9.76*	*0.122*	*0.112*	*0.789*
FCR_2, g:g	1.19	1.18	1.17	1.15	*0.028*	*0.843*	*0.055*	*0.965*

Abbreviations: CON+: positive control diet; CON-: negative control diet; CON+EW: positive control diet supplemented with 1% of earthworm; CON+VC: positive control diet supplemented with 1% vermicompost; CON-EW: negative control diet supplemented with 1% earthworm; CON-VC: negative control diet supplemented with 1% vermicompost; CV in BW: coefficient of variation in body weight of the birds in a pen (%); BW: average body weight in a pen; WG: average weight gain in a period; ADG: average daily gain in a period; Feed intake: Average total feed intake per bird in a given period, g/ bird; FCR_1: Feed:weight gain ratio, g/g; DMI_feed; dry matter intake of birds in a given period, g/bird; DMI_feed: dry matter intake through feed, g/bird; DMI_EW: DM intake through EW consumption; DMI: total dry matter intake, g/bird; FCR_2: DMI to weight gain ratio, g/g; DxB: Diet and Batch interaction.

n.a. = not applicable.

a,b Groups denoted with different letters differ significantly (Tukey, *P* < 0.05).

†,‡ Indicates tendency of 2 diets to differ (Tukey, *P* = 0.103 and *P* = 0.063, respectively). Data are presented as LSEMANS and their SE. In the interest of a clear presentation, only the most conservative (i.e., the largest) SE is presented. Since the n numbers for all groups were the same in the second period (n = 6), SEs of LSMEANS were identical for this period.

In P2, CON- did not affect broiler growth or DMI as compared to CON+ (*P* > 0.05), although CON- birds consumed approximately 6% higher amounts of feed than those birds on the CON+ diet. In the same period (P2), CON-VC fed birds consumed a numerically (*P* = 0.106) higher amount of feed (about + 7%), tended to have a higher ADG (*P* = 0.084) and were still heavier (*P* < 0.05) than those birds fed on CON+. Birds on CON-EW did not differ (*P* > 0.05) from those on CON- or CON-VC diets in terms of growth, feed intake and feed conversion efficiency parameters.

### Earthworm Acceptance and Consumption by Broilers

**Figure 2:**
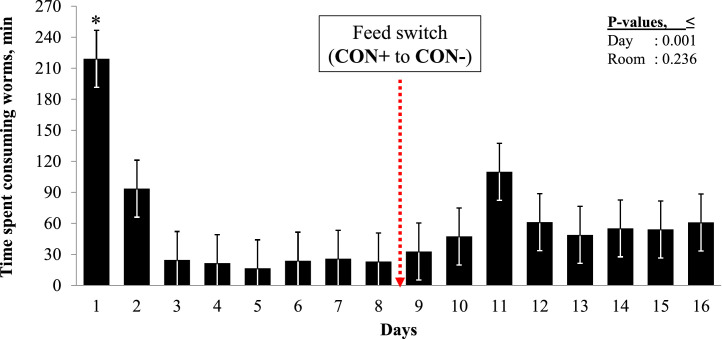
Time-spent eating earthworms by the birds of Group 3 over the experimental days. *: Significantly different from all the other days (Tukey, *P* < 0.05). Each bar represents average of 6 pens on a given day. Note that number of earthworm consuming birds in each pen was n = 10 birds while feeding on CON+ diet in period 1 (d1–d8), and n = 5 birds while feeding on CON- diet in period (d9–d16).

Average daily amount of fresh EW consumed by the birds in a pen ranged from 0.36 to 4.8 g per bird (mean = 2.6 g; SD = 1.49) from d1 to d16. Overall average time spent consuming EW was approximately 49 min (SD = 81). Except for d1, there was no significant difference in time spent consuming EW across the 16 d (Figure 2).

### Litter Moisture and Pasty vent

**Figure 3:**
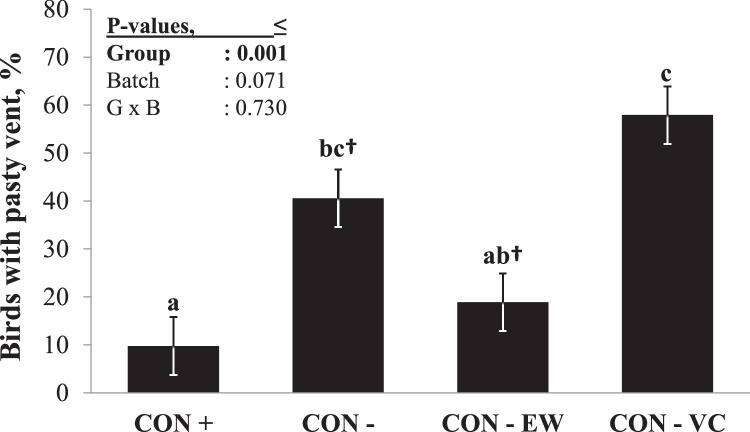
Average incidence of pasty vent in the end of second period in broilers fed different diets. abc: groups denoted with different letters differs significantly (Tukey. *P* < 0.05). ^†^: Groups sharing the sign tend to differ (Tukey. *P* = 0.072). Replicates: A pen was considered as replicate (N = 48. i.e., 6 pens per group in each of 2 batches). Raw data: incidence of birds with pasty vent during the last 6 d of the experiment was averaged for each pen.

The parameters used to assess moisture content and accumulation in the litter showed no differences across the experimental diets in either period (*P* > 0.05; Supplementary Table 3). Although proportion of moisture accumulated in the litter was numerically lowest in CON+ (6.2%) and highest in CON-VC (8.0%), the differences were not significant until the end of P2 (*P* > 0.05). During the last 6 d of P2, 10% of CON+ birds had PV (Figure 3). As compared to CON+, the CON- diet (*P* < 0.05) increased the incidence of PV (40.5%), and VC supplementation aggravated this effect (57.9%), whereas CON-EW (18.9%) did not differ from CON+ (*P* > 0.05). As compared with CON-, CON-EW tended to decrease (*P* = 0.072) the incidence of PV. Similarly, birds fed on CON-EW had a lower incidence of PV as compared to those birds on CON-CV (*P* < 0.05).

### Plasma Concentrations of Immunoglobulin Isotypes, Albumin, Globulin, and Total Protein

Supplementing the CON+ diet with earthworms (i.e., CON+EW) tended to increase plasma IgM levels compared to CON+ without earthworms (*P* = 0.080, Table 4) in P1, while no differences were observed for IgY and IgA, and the other parameters measured in the same period (*P* > 0.05). In P2, however, CON-VC tended to result in higher IgM levels than did CON-EW (*P* = 0.064). Plasma albumin concentration was higher in birds fed on CON-VC than in those birds fed on CON+ or CON-EW (*P* < 0.05). Similarly, plasma concentrations of globulin and total protein were higher in CON-VC fed birds than in CON+EW fed birds (*P* < 0.05).

**Table 4:** Dietary effects on concentrations of plasma immunoglobulin isotypes, albumin, globulin and total protein in broiler birds fed different diets in 2 consecutive periods.

	Diets					
Period 1 (d8)	CON+	CON+	CON+EW	CON+VC	*SE*	*P-value*
IgY, µg/mL	943.0	-	880.1	889.9	57.5	0.624
IgA, µg/mL	69.4	-	98.9	73.3	18.0	0.393
IgM, µg/mL	57.8^†^	-	92.5^†^	61.4	12.8	0.080
Albumin, mg/mL	9.9	-	10.1	10.2	0.30	0.595
Globulin, mg/mL	13.8	-	14.3	14.5	0.79	0.768
Total protein, mg/mL	23.7	-	24.2	24.7	0.98	0.687
**Period 2 (d16)**	**CON+**	**CON-**	**CON-EW**	**CON-VC**		
IgY, µg/mL	1058.8	984.5	874.2	1443.7	245.2	0.332
IgA, µg/mL	253.3	258.6	254.4	328.5	38.8	0.396
IgM, µg/mL	142.5	139.7	114.1^†^	205.5^†^	25.9	0.064
Albumin, mg/mL	10.6^a^	10.9^ab^	10.3^b^	11.5^a^	0.21	0.001
Globulin, mg/mL	13.8^ab^^†^	14.3^ab^	12.7^b^	15.9^a^^†^	0.63	0.004
Protein, mg/mL	24.4^ab^^†^	25.2^ab^	23.0^a^	27.4^b^^†^	0.79	0.001

a,b Groups denoted with different letters differ significantly (Tukey, *P* < 0.05 ^†^: Tukey, *P* < 0.10). Data are presented as LSMEANS and their SE. In the interest of a clear presentation, only the most conservative SE (i.e. the largest) is presented. Abbreviations: CON+: positive control diet; CON-: negative control diet; CON+EW: positive control diet supplemented with 1% of earthworm; CON+VC: positive control diet supplemented with 1% vermicompost; CON-EW: negative control diet supplemented with 1% earthworm; CON-VC: negative control diet supplemented with 1% vermicompost.

### Small Intestine and Ceca Weights

Earthworm consumption tended to increase (*P* = 0.081) the proportion of small intestine (i.e., JI-ResEYS) to BW ratio, with no significant effect on the weight of JI-ResEYS (*P* = 0.491) in P1 (Table 5). Compared to CON+, CON+VC tended to increase (*P* = 0.054) cecal weight in P1, which then disappeared in P2 when they switched to the CON-VC diet (*P* > 0.05). In contrast, the cecal weight of birds consuming EW did not differ from birds fed CON+ in P1 (i.e., CON+EW), but cecal weight was increased (*P* = 0.031) compared to CON+, when they switched to the CON-EW diet in P2. CON- had no significant effect on JI-ResEYS or cecal weights when fed alone (i.e., CON-) nor when supplemented with vermicompost (i.e., CON-VC) in P2 (*P* < 0.05).

**Table 5:** Absolute weights and proportions of small intestine (jejunum + ileum + residual egg yolk sac; JI-ResEYS) and ceca to body weight, as well as genomic DNA of the host (CYTB DNA) and bacteria (16S DNA) in colon samples of broilers fed different diets.

	Diets					*P-values*		
Period 1 (d8)	CON+	CON-	CON+EW	CON+VC	*SE*	*Diet*	*Batch*	*DxB*
JI-ResEYS, g	15.57	n.a.	16.08	16.10	*0.441*	*0.491*	*0.031*	*0.358*
JI-ResEYS, % of BW	7.75	n.a.	8.12	7.74	*0.143*	*0.081*	*0.127*	*0.244*
Ceca, g	2.57	n.a.	2.69	2.91	*0.118*	*0.054*	*0.809*	*0.816*
Ceca, % of BW	1.28	n.a.	1.37	1.42	*0.055*	*0.114*	*0.237*	*0.671*
CYTB DNA	0.091	n.a.	0.118	0.110	n.a.	*0.834*	n.a.	n.a.
16S rDNA	0.084	n.a.	0.090	0.118	n.a.	*0.915*	n.a.	n.a.
**Period 2 (d16)**	**CON+**	**CON-**	**CON-EW**	**CON-VC**				
JI-ResEYS, g	36.59	39.16	37.73	39.90	*1.274*	*0.249*	*0.137*	*0.357*
JI-ResEYS, % of BW	6.56	6.63	6.54	6.24	*0.252*	*0.690*	*0.099*	*0.763*
Ceca, g	6.57^a^	6.90^ab^	7.73^b^	7.36^ab^	*0.288*	*0.031*	*0.001*	*0.377*
Ceca, % of BW	1.18	1.18	1.33	1.20	*0.061*	*0.256*	*0.071*	*0.693*
CYTB DNA	0.150	0.086	0.100	0.067	n.a.	*0.263*	n.a.	n.a.
16S rDNA	0.115^x^	0.043^y^	0.077^xy^	0.075^xy^	n.a.	*0.049*	n.a.	n.a.

Abbreviations: CON+: positive control diet; CON-: negative control diet; CON+EW: positive control diet supplemented with 1% of earthworm; CON+VC: positive control diet supplemented with 1% vermicompost; CON-EW: negative control diet supplemented with 1% earthworm; CON-VC: negative control diet supplemented with 1% vermicompost.

n.a. = not applicable as the CON- diet was fed only in P2.

a,b Groups denoted with different letters differ significantly (Tukey, *P* < 0.05).

x,y Groups denoted with different letters differ significantly (Dunn's test, *P* < 0.05; following a significant exact p-value of the Kruskal–Wallis test with Monte Carlo estimate, *P* = 0.049). Except for CYTB DNA and 16S rDNA, data are presented as LSMEANS and their standard errors (**SE**). For the sake of a succinct presentation, only the most conservative SE (i.e., the largest) is presented for each variable. CYTB DNA and 16S DNA data are presented as median values. Since the n numbers replicates for all groups were the same in the second period (n = 6). SEs of LSMEANS were identical for this period.

### Host and Microbial Genomic DNA in Host Feces

No dietary effects on host DNA or bacterial 16S rDNA were observed in colon contents in P1 (*P* > 0.05, Table 5). There were also no differences among the 4 groups in host DNA contents of colon samples collected at d 16 (*P* = 0.263), whereas CON- lowered 16S rDNA content as compared with CON+ (*P* < 0.05). However, supplementation of CON- with EW or VC resulted in no differences in colon 16S rDNA contents (*P* > 0.05) when compared with the positive control diet (CON+) in P2. There were no significant correlations of bacterial 16S rDNA with FCR or DMI observed in either period (*P* > 0.05, Table 6). Correlations between host and bacterial DNA contents were also non-significant in both periods (*P* > 0.05). However, there was a significant positive correlation (*r* = 0.45, *P* < 0.05) between FCR and CYTB DNA content in the colon contents in P1, which was no longer significant in P2 (*P* > 0.05). Although there was no correlation between CYTB DNA and DMI in P1 (*r* = 0.02, *P* > 0.05), there was a significant negative correlation (*r* = -0.47, *P* < 0.05) between these 2 variables in P2.

**Table 6:** Correlations between dry matter intake (**DMI**), feed conversion ratio (**FCR**) and genomic DNA of host (CYTB DNA) and bacteria (16S DNA) in colon samples of broilers in period 1 (upper diagonal) and period 2 (lower diagonal).

	DMI	FCR	CYTB DNA	16S DNA
DMI	n.a.	0.22	−0.02	−0.26
FCR	0.21	n.a.	0.45⁎	−0.17
CYTB DNA	−0.47⁎	−0.22	n.a.	−0.09
16S DNA	−0.01	0.29	−0.19	n.a.

Abbreviation: n.a., not applicable.

⁎ The sign represents coefficients of correlation that are significantly different from zero (*P* < 0.05). Pooled data across 4 groups within each period were used for the correlation analysis. Number of observations, n = 24 pens/period (i.e., N = 48 pens in 2 periods). DMI and FCR are direct pen averages, whereas CYTB DNA and 16S DNA are based on average of 2 to 3 individual bird measurements from each pen. DMI represents dry matter intake through feed and earthworm in a period, and FCR represents DMI per gram body weight gain.

## DISCUSSION

Overall, the performance data from the 2 identical batches of the experiment collectively indicate that feeding of EW (1%) had no detrimental effect on growth performance or feed intake of the birds. The addition of VC to the feed at 1% of DM increased feed intake and thereby improved broiler growth in the first period, but these effects weakened in the second period when VC was supplemented to the negative control diet (**CON-**). The challenge diet (**CON-**) did not impair bird performance during the short 8-d period, but decreased proportion of bacterial 16S rDNA in colon contents, and increased incidence of pasty vent, presumably due to NSP-associated anti-nutritive effects. Feeding EW to the birds increased cecal size and tended to reduce the incidence of PV associated with the CON- diet, which may indicate that inoculation with beneficial microbiota through EW might have reduced the anti-nutritive effects of NSP.

The positive correlation between FCR and genomic host DNA content in colon contents supports the hypothesis that feed efficiency in broilers might be negatively associated with gut cell losses, which nevertheless held true only for the first period of the experiment when a standard control diet was fed to the birds. In the following sections, we briefly address the reproducibility of the results presented, discuss potential mechanisms by which dietary EW supplementation mitigates NSP-associated anti-nutritive effects, and conclude with potential implications.

### Reproducibility of the Results

Reproducibility of the performance data, which were measured over 2 identical experimental batches, can be assessed through absence and presence of interactions between dietary treatments and experimental batch. As shown in Table 3, Table 5, there were significant effects of experimental batch on some of the growth, feed intake and intestinal organ size parameters, which were higher in B2 than in B1 (data not shown). However, since there was no significant interaction between treatment and batch, all these parameters were influenced by the batch effects similarly, implying reproducibility of the treatment effects over the 2 experimental batches. Probably one of the most important reasons for the greater performance parameters in the second batch was the initial BW of the birds on arrival day (i.e., 38.8 vs. 42.6 g/bird in B1 and B2, respectively), an influential factor for post-hatch growth with diminishing time-dependent effects up to 2 to 3 wk of life (Pinchasov, 1991; Wilson, 1991).

### Effects of the NSP-Enriched Challenge Diet

Although chickens are able to increase their feed intake by up to 10% when offered energy and nutrient diluted diets (i.e., insoluble NSP-rich diets) (Daş et al., 2014), both CON+ and CON- diets without or with VC had very similar nutrient and energy contents, while different ingredients were used to produce the NSP-enriched challenge diet (CON-) through inclusion of barley, wheat and rye at the expense of corn. Compared to the positive control diet (CON+), the NSP-enriched CON- diet contained 34% and 7% more soluble (S-) and insoluble (I-) NSP, respectively, with mannose, xylose, glucose and arabinose accounting for the largest proportional differences between the 2 diets. Since no NSP-degrading enzymes were added to the diet, it was expected that the CON- diet would induce NSP-associated anti-nutritive effects, as the amount of soluble NSP (**S-NSP**) in a diet determines the degree of anti-nutritive effects in chickens (Choct, 1999). S-NSP can considerably increase the viscosity (Nguyen et al., 2021), likely contributing to the production of sticky feces and potentially explaining the higher incidence of PV in birds fed the CON- diet. Although the CON- diet increased the incidence of PV, there was no effect on litter moisture content, which could be related to the smaller size of the birds, that only stayed in the pens for a limited period of time (8d), resulting in a negligible amount of feces accumulating in the pens. Similarly, CON- did not affect feed intake and bird growth performance during the short 8-d period (P2). Although it is well known that high levels of S-NSP can reduce digestibility of macronutrients (Smits and Annison, 1996), feed conversion efficiency was not adversely affected by the CON-diet. This probably indicates an insufficient level of S-NSP in CON- diet to affect digestion within the short period of 8 d.

Along with the increased incidence of PV, the most noticeable effect of the CON- diet was a reduced proportion of bacterial 16S rDNA in the colon contents, with no measurable impact on colonic host DNA. We used host DNA content in colonic digesta as a marker for intestinal cell losses as suggested for pigs (Slinger et al., 2019) and chickens (Slinger et al., 2020). Indeed, the positive correlation between FCR and genomic host DNA in colon samples supports the hypothesis that feed efficiency in broilers might be negatively associated with gut cell losses, which nevertheless held true only in P1 when all the birds were fed on CON+ diets without or with EW and VC, but not in P2 when they were switched to CON- diets. However, the absence of a significant correlation in P2 may not only be attributed to the CON- diet, as age-related differences in host DNA content of colon samples between P1 and P2 cannot be excluded. The lower proportion of bacterial 16S rDNA in CON- birds in P2 is nevertheless fully attributable to the dietary effects, and may indicate alterations in the microbial communities as a result of the switch from the CON+ to the NSP-enriched CON- diet, which is expected to modulate gastrointestinal environment and gut microbiota in chickens (Nguyen et al., 2021). The lower proportion of bacterial 16S rDNA induced by the NSP-enriched CON- diet is however in contradiction with previous results, as S-NSP supplemented diets are expected to fuel microbial species (Nguyen et al., 2021). A possible explanation for the reduced abundance of microbial DNA might be associated with re-establishment of bacterial communities in the intestinal tract, that is, microorganisms that are able to survive and reproduce in an NSP-induced environment after the diet switch might not yet be fully abundant in a short period of 8 d. Furthermore, microorganisms under NSP influences might have better adhered to the gut wall, thereby resulting in less microbes being present in the colon contents. Thus further investigation on the microbiota composition of colonic samples is warranted.

### Effects of Vermicompost and Earthworms

Earthworms have previously been considered as an animal protein source for poultry (Tagoba, 1980; Reinecke et al., 1991) because of their high CP content (Table 1) and well-balanced amino acid composition (Supplementary Table 1), while recent studies focused more on feed additive properties of EW (e.g., as prebiotic) at low dietary inclusion levels (Bahadori et al., 2017; Sun et al., 2020). Supplementation of 1% VC to the CON+ diet (i.e., CON+VC) improved bird growth through increased feed intake (5.7% relative to CON+), while no effect on FCR was observed in P1. The increase in feed intake was even higher in P2 (7.7%) when VC was supplemented to the CON- diet. However, this second increase in the feed intake did not seem to be directly induced through VC-supplementation, but mainly due to the CON- diet itself, with the birds in P2 consuming approximately 6.2% more feed than CON+. These results indicate that VC stimulates feed intake of birds when supplemented to a standard control diet (CON+), but not in birds on the CON- diet which had already increased their feed intake. The experimental design did not include paired control groups in a cross-over format, which would have involved feeding CON+VC (and CON+EW) in P2. As a result, it remains unclear whether feeding VC would continue to improve feed intake and growth performance in the second period of the experiment.

Provision of EW to the birds did not alter feed intake or growth performance in either period, implying that feeding EW had no adverse effect on the birds’ performance. One of the most noticeable and favorable effects of EW supplementation to the CON- diet was the lower frequency of PV. According to Fujii et al. (2012) the intestine of EW contains microorganism with exocellulase and xylanase activities, which may be associated with lignocellulose decomposition activity of EW under natural circumstances. This implies that feeding of EW may improve fecal consistency, presumably through its effect as a microbiota inoculant. Whether EW supplementation contributed to increased NSP-degrading enzyme activity in the chicken gut needs to be specifically investigated in further studies covering potential changes in the microbiota. A potentially increased NSP-degrading activity might also be attributed to enzyme secretions from the earthworms (Fujii et al., 2012). It is interesting to note that EW decreased the incidence of PV while VC did the opposite. It is reasonable to assume that PV-reducing effects could be expected by feeding VC, too, as vermicompost is basically composed of worm feces, which might be expected to have a similar microbiota as the EW. As summarized in Supplementary Table 2, supplementation of VC to the positive (CON+VC) or negative control diets (CON-VC) increased microbial loads of these 2 diets as compared to the respective diets without VC (i.e., CON+ and CON-) for microorganism groups of 1 and 2, leading to higher field-borne and product typical, and spoilage indicator microorganisms (e.g., among others *Bacillus* spp., *Staphylococcus, Micrococcus*), which were present even in much higher quantitates in EW. Although the existence of some feed-borne pathogens (e.g. *Salmonella*) are linked with their colonization in the gastrointestinal tract, much less is known about non-pathogenic feed microorganisms and their impact on the gastrointestinal tract microbiota and functions (Olson et al., 2020). Assuming that EW and VC may alter microbial composition of the gut and thereby affect fecal consistency, the question arises why EW and VC led to opposite effects on the incidence of PV. This might be related to the viability of the microorganisms present in EW and VC as well as presence of active enzymes in EW. It should be noted that EW were always fed to the chicks fresh, whereas VC was included in the feed before the experiment started, implying the time from addition of VC to the feed until its consumption (2–3 wk) might have influenced the viability or the microbial composition of VC.

It is known that the gut microbiota can mediate the anti-nutritive effects of soluble and viscous NSPs (Smits and Annison, 1996). It is worthy of note that EW increased cecal weight by 18% relative to CON+, when given on top of the CON- diet in P2. Cecal microbiota of chickens enable them to ferment various fibers (Dunkley et al., 2007), which in turn modulates the gastrointestinal tract environment (Nguyen et al., 2021). The higher cecal weight can be considered as an indication of increased bacterial activity, which is associated with increased production of short chain fatty acids that can be rapidly absorbed by the epithelial cells, and serve as a source of energy, leading to stimulation of epithelial cell proliferation and thus may explain the enlarged cecal size (Sudo and Duke, 1980; Montagne et al., 2003; Józefiak et al., 2004). The CON+VC diet also tended to increase cecal size in P1, but this effect disappeared in P2 with CON-VC, although cecal weight was still 12% higher for CON-VC than with CON+.

Intestinal microbiota play an important role in the development of the immune system (Haghighi et al., 2006; Zenner et al., 2021). Bahadori et al. (2017) reported increased lactic acid bacteria and reduced *E. coli* counts in ceca of broilers fed diets supplemented with EW meal and VC. A microbiota enriched with beneficial microorganisms in the chicken gut can lead to considerable interactions with the immune system, particularly through stimulation of the adaptive immune responses, which are associated with the production of natural antibodies (Pan and Yu, 2014). For instance, day-old-chicks fed probiotics exhibited elevated levels of natural antibodies reactive to different antigen levels (e.g., tetanus toxoid and bovine serum albumin) in serum and/or intestines (Haghighi et al. 2006). Development of the immune system in chickens is substantially affected by early microbial colonization of the GIT. Zenner et al. (2021) found increased levels of plasma IgY and IgA in chickens inoculated with maternal fecal microbiota after hatch, as compared to control birds kept under specific-pathogen free conditions. In poultry, IgM is the predominant Ig-isotype after exposure to a novel antigen in the primary antibody response, while IgY is the main isotype produced in the secondary systemic antibody response (Härtle et al., 2022). In the present study we observed trends, indicative of a stimulated primary antibody response as indicated by elevated levels of IgM after feeding EW (in P1) and VC (in P2). Elevated plasma concentrations of albumin, globulin and total protein in CON-VC fed birds as compared to those in CON+ fed birds, supports the hypothesis that VC could have an even stronger immune stimulatory effects than EW. Immune-stimulating effects of EW and VC reported in this study are in line with the humoral immune responses of chickens reported by Bahadori et al. (2017), who showed increased levels of vaccine-induced antibody titers against Avian Influenza in birds fed diets supplemented with EW meal or VC.

### Potential Broiler Welfare Implications and Biosafety Risks with Feeding Earthworms

Broiler chickens are considered an extremely inactive strain of the chicken species, and their behavioral patterns differ significantly from their Jungle fowl ancestors (Weeks et al., 2000). For example, healthy broilers spend about 76% of their time lying, while they spend only 3% pecking on the ground, which is much less than that of Red Jungle fowl (Dawkins, 1989; Weeks, et al., 2000). Compared with layer type chickens, broilers spend less time eating (Masic et al., 1974) and are less active (Lindqvist et al., 2006), likely due to the relatively higher energetic cost of locomotion that increases disproportionally with increasing BW as they get older (Tickle et al., 2018). Similar to feeding of insect larvae to chickens, provision of EW may be considered as an edible environmental enrichment for broilers to increase their activity and thereby improve animal welfare (Pichova ete al., 2016; Riber et al., 2018). Environmental enrichment alleviates the monotony of barren environments, enabling birds to express natural behaviors. It also helps reduce stress and fear, and supports the development of cognitive functions such as learning and memory (Tahamtani et al., 2018). After a short period of learning (1–2 d; Figure 2), EW-consuming birds spent approximately 50 min to consume 1% of their DMI as fresh EW, which corresponded to approximately 6.7% of their fresh matter intake. During the availability of the EW in the pens, the birds exhibited several species-specific behaviors, including exploration of EW as novel objects, ground foraging, and food-running (Supplementary Video 1), which are rarely observed in barren environments. A concern about feeding of EW or insect larvae to chickens is the increased flock heterogeneity in BW of the birds, which might be caused by selective feeding and competition of the birds for the readily consumed edible materials. At the present dietary inclusion level (1% DM), provision of EW to the birds did not negatively influence BW uniformity, which is in line with the recent data on inclusion of black soldier fly larvae (BSFL) up to 20% in broiler rations, whereas 30% inclusion of BSFL in the ration led to a larger CV in the BW of the birds (Seyedalmoosavi et al., 2022).

From a biosafety perspective, both EW and VC cannot be considered completely safe for chickens as they may contain pathogenic organisms, although both EW and VC samples used in the present study were shown to be negative for *Salmonella* spp. (Supplementary Table 2). It is known that EW collected from fields can serve as paratenic host or vectors for the transmission of certain parasitic pathogens (i.e., eggs of *Heterakis gallinarum* and thus *Histomonas meleagridis*) in chickens (Cupo and Beckstead, 2019), whereas EW raised under controlled environmental conditions may be specific pathogen free. Thus the source of both EW and VC is of crucial importance, i.e. they should be free of pathogens known to induce infections in chickens.

It is concluded that the challenge diet (CON-) diet did not impair broiler performance, but increased incidence of PV and decreased proportion of 16S rDNA in host colon samples likely due to the anti-nutritive effects induced by NSP. Vermicompost supplementation to the standard control diet increased feed intake and thereby improved growth in the first week of life. Feeding of EW to the birds had no negative effects on performance, but reduced the incidence of PV and increased cecal size, indicating potential inoculation of GIT with beneficial microorganisms that help to mitigate anti-nutritive effects of NSP. The addition of VC and EW may induce immune stimulatory effects in the birds, while the provision of EW to broilers may be considered as environmental enrichment to improve animal welfare.

## DISCLOSURES

The authors declare no conflicts of interest.

## Appendix Supplementary materials

Image, application 1

Image, video 2

## Footnotes

Supplementary material associated with this article can be found in the online version at doi:10.1016/j.psj.2024.104341.
