# Supplementary material for: Effects of feeding earthworm or vermicompost on early life performance of broilers under challenging dietary conditions
Source: Poult Sci. 2024 Sep 18;103(12):104341. doi: 10.1016/j.psj.2024.104341 (PMC11490703; doi:10.1016/j.psj.2024.104341)
Supplement: Supplementary file 1 [file mmc1.docx]

**Supplementary Table 1.** Amino acid (AA) composition of the experimental diets and earthworms.

|  | **Experimental diets** | | | |  | |
| --- | --- | --- | --- | --- | --- | --- |
| **AA (mg/g DM)** | **CON+** | **CON-** | **CON+VC** | **CON-VC** | | **EW** |
| **Essential** |  |  |  |  | |  |
| Arginine | 15.8 | 15.8 | 16.1 | 15.3 | | 44.8 |
| Histidine | 6.0 | 5.9 | 6.14 | 5.6 | | 14.6 |
| Isoleucine | 9.6 | 9.6 | 9.9 | 9.3 | | 25.4 |
| Leucine | 20.3 | 19.2 | 20.4 | 18.5 | | 48.9 |
| Lysine | 15.9 | 17.0 | 16.8 | 16.1 | | 47.3 |
| Methionine^1^ | 5.4 | 5.4 | 5.9 | 5.9 | | 9.1 |
| Phenylalanine | 11.5 | 11.6 | 11.7 | 11.4 | | 23.8 |
| Threonine | 10.1 | 10.4 | 10.5 | 10.7 | | 29.8 |
| Valine | 13.8 | 11.5 | 11.4 | 11.3 | | 28.0 |
| **Non- essential** |  |  |  |  | |  |
| Aspartic acid + Asparagine | 25.5 | 25.6 | 26.1 | 25.3 | | 64.2 |
| Glutamic acid + Glutamine | 45.8 | 48.8 | 46.2 | 46.9 | | 93.0 |
| Cysteine^2^ | 0.2 | 0.1 | 0.1 | 0.2 | | 2.1 |
| Serine | 12.6 | 12.6 | 12.8 | 14.3 | | 33.3 |
| Glycine | 10.2 | 10.4 | 10.5 | 10.6 | | 37.8 |
| Alanine | 11.8 | 11.0 | 11.8 | 11.2 | | 33.7 |
| Tyrosine | 6.2 | 5.9 | 6.1 | 6.2 | | 16.8 |
| Proline | 12.4 | 13.6 | 12.5 | 13.3 | | 22.7 |
| **Sum of AA**^3^ | **233.2** | **234.4** | **234.7** | **232.1** | | **575.3** |

^1^ Methionine was determined as the sum of methionine and its oxidation products methionine sulfoxide and methionine sulfone.

^2^ Cysteine was partly oxidized during hydrolysis, and is thus likely underestimated.

^3^ Represents sum of free- and protein-bound amino acids.

**Abbreviations**: **CON+**: positive control diet; **CON-**: negative control diet; **CON+VC**: positive control diet supplemented with 1% vermicompost; **CON-VC**: negative control diet supplemented with 1% vermicompost; **EW**: earthworm.

**Supplementary Table 2.** Assessment of the microbial quality of feed and earthworm samples by an accredited laboratory***^1^*** using standard VDLUFA methods***^2^***. Figures presented in the table are colony-forming units (10^3^ cfu/g fresh sample).

| **Microorganism groups (MOG)*^3^*** | **Experimental diets** | | | | | | | |  | |
| --- | --- | --- | --- | --- | --- | --- | --- | --- | --- | --- |
|  | **CON+** | | **CON-** | | **CON+VC** | | **CON-VC** | | **Earthworm** | |
|  | S1 | S2 | S1 | S2 | S1 | S2 | S1 | S2 | S1 | S2 |
| MOG 1: Field-borne / typical for product | 14 | 16 | 78 | 300 | 160 | 210 | 150 | 465 | 3500 | 3400 |
| MOG 2: Spoilage indicator | 5 | 3 | 5 | 20 | 11 | 10 | 35 | 6 | 25 | 40 |
| MOG 3: Spoilage indicator | < 1 | < 1 | < 1 | < 1 | 3 | 5 | 6 | 6 | < 1 | < 10 |
| MOG 4: Field-borne / product-typical | 33 | < 1 | 6 | < 1 | 10 | 3 | 2 | < 1 | 2 | 4 |
| MOG 5: Spoilage indicator (storage molds) | < 1 | < 1 | 10 | < 1 | 1 | 3 | 2 | 3 | 1 | 2 |
| MOG 6: Spoilage indicator (mucorales molds) | < 1 | < 1 | < 1 | < 1 | 1 | 1 | 1 | 1 | 2 | 1 |
| MOG 7: Spoilage indicator (yeasts) | < 1 | < 1 | < 1 | 8 | < 1 | < 1 | < 1 | 4 | 3 | 2 |
| *Salmonella* spp. (in 25 g) | n.d. | n.d. | n.d. | n.d. | n.d. | n.d. | n.d. | n.d. | n.d. | n.d. |
| **Overall quality class*^4^*** | **2** | **1** | **1** | **1** | **1** | **1** | **1** | **1** | **n.a.** | **n.a.** |

**Abbreviations**: **CON+**: positive control diet; **CON-**: negative control diet; **CON+VC**: positive control diet supplemented with 1% vermicompost; **CON-VC**: negative control diet supplemented with 1% vermicompost; **S1**: sample #1; **S2**: sample #2; **n.d.**: not detectable; **n.a.**: no official assessment was made by the lab as earthworms are not considered typical feed samples.

***^1^*** Analyses were performed by the feed laboratory of Landwirtschaftliche Untersuchungs-und Forschungsanstalt, LMS Agrarberatung GmbH (Rostock, Germany).

***^2^*** VDLUFA-Methodenbuch Band III, Die chemische Untersuchung von Futtermitteln. Chapter 28.1. Microbiological methods, VDLUFA-Verlag, Darmstadt, Germany. Available online at <https://www.vdlufa.de/Methodenbuch/index.php?option=com_content&view=article&id=4&Itemid=111&lang=de>

***^3^***Microorganism considered in the **groups** are as following; **MOG-1**: Yellow pigmented bacteria (*Erwinia* spp.), *Pseudomonas*/*Enterobacteriaceae*, saprophytic *coryneform* bacteria; **MOG-2**: Indicator microorganisms: *Bacillus* spp., *Staphylococcus*, *Micrococcus*; **MOG-3**: Indicator microorganisms: *Streptomycetes*; **MOG-4**: Saprophytic molds (field molds): *Dematiaceae, Verticillium* spp., *Acremonium* spp., *Fusarium* spp., *Aerobasidium* spp; **MOG-5**: Indicator microorganisms (storage molds): *Aspergillus* spp., *Penicillium* spp., *Scopulariopsis* spp., *Wallemia* spp. **MOG-6**: Indicator microorganisms: Mucorales molds (*Mucor* spp., *Rhizopus* spp.); **MOG-7**: Indicator microorganisms: Yeasts (*Candida*, *Rhodontorula*). For a detailed description of the **MOGs** in English the reader is referred to Nešić, et al. (2020).

***^4^*** There are 4 **quality classes** described by VDLUFA using microbiological loads represented in MOGs. **Class 1**: desirable; **Class 2**: reduced quality; **Class3**: poor quality; **Class-4**: feed is not acceptable. Feeds to be used in animal nutrition should be in overall quality classes 1 to 3. Feed classified in quality class 4 is rated as not suitable for animal feeding (Nešić, et al., 2020).

**Reference cited**: Nešić, K., Pavlović, M., & Ivanović, S., 2020. Assessment of the microbiological quality of feed using the Verbands Deutscher landwirdschaftlicher Untersuchungs und Forschungsanstalten (VDLUFA) method. *Veterinarski Glasnik*, *74*(1), 68-76. <https://doi.org/10.2298/VETGL190710016N>

**Supplementary Table 3**. Dry matter, moisture content and moisture accumulated in the litter samples collected from pens of birds receiving different diets.

|  | **Diets** | | | | ***SE*** | ***P-values*** | | |  |  |
| --- | --- | --- | --- | --- | --- | --- | --- | --- | --- | --- |
| **Period 1 (d-1d8)** | **CON+** | **CON-** | **CON+EW** | **CON+VC** |  | ***Diet*** | ***Batch*** | ***DxB*** |  |  |
| Dry mater, % | 90.2 | n/a | 90.2 | 88.7 | *1.15* | *0.516* | *0.559* | *0.502* |  |  |
| Moisture, % | 9.8 | n/a | 9.8 | 11.3 | *1.15* | *0.516* | *0.559* | *0.502* |  |  |
| Moisture accumulated, % | 7.2 | n/a | 7.3 | 8.8 | *1.15* | *0.516* | *0.559* | *0.502* |  |  |
| **Period 2 (d1-d16)*** | **CON+** | **CON-** | **CON-EW** | **CON-VC** |  |  |  |  | |  |
| Dry mater, % | 91.3 | 90.5 | 90.1 | 89.5 | *1.01* | *0.628* | *0.308* | *0.566* |  |  |
| Moisture, % | 8.7 | 9.5 | 9.9 | 10.5 | *1.01* | *0.628* | *0.308* | *0.566* |  |  |
| Moisture accumulated, % | 6.2 | 6.9 | 7.4 | 8.0 | *1.01* | *0.628* | *0.308* | *0.566* |  |  |

Basal dry matter of the clean, unused litter material (i.e. wood shaving) was 97.4% at the beginning of the experiment. Moisture accumulated in the litter until end of a period was calculated as the difference of dry matter in basal sample and the sampled litter in the end of each period.

*****: Note that used litter was not replaced with fresh litter in the end P1, thus moisture contents in the end of P2 reflect an accumulation of water from beginning of P1 to the end of P2 (i.e. d1-d16).

**Abbreviations**: ; **CON-**: negative control diet; **CON+VC**: positive control diet supplemented with 1% vermicompost; **CON-VC**: negative control diet supplemented with 1% vermicompost; **CON-EW**: negative control diet supplemented with 1% earthworm. **DxB**: Diet and Batch interaction. **n/a** = not applicable as the CON- diet was fed only in P2.

**Replicates**: Pen was considered as replicate (N=48). Except for CON+ diet in period 1 (n=12), numbers of pens per diet was n=6 in both periods 1 and 2, respectively.
